# How to Measure Behavioral Spillovers: A Methodological Review and Checklist

**DOI:** 10.3389/fpsyg.2019.00342

**Published:** 2019-04-05

**Authors:** Matteo M. Galizzi, Lorraine Whitmarsh

**Affiliations:** ^1^London School of Economics and Political Science, Department of Psychological and Behavioural Science, London, United Kingdom; ^2^School of Psychology, Cardiff University, Cardiff, United Kingdom

**Keywords:** spillovers, mixed-methods, experimental design, lab-field experiments, behavioral spillovers

## Abstract

A growing stream of literature at the interface between economics and psychology is currently investigating ‘behavioral spillovers’ in (and across) different domains, including health, environmental, and pro-social behaviors. A variety of empirical methods have been used to measure behavioral spillovers to date, from qualitative self-reports to statistical/econometric analyses, from online and lab experiments to field experiments. The aim of this paper is to critically review the main experimental and non-experimental methods to measure behavioral spillovers to date, and to discuss their methodological strengths and weaknesses. A consensus mixed-method approach is then discussed which uses between-subjects randomization and behavioral observations together with qualitative self-reports in a longitudinal design in order to follow up subjects over time. In particular, participants to an experiment are randomly assigned to a treatment group where a behavioral intervention takes place to target behavior 1, or to a control group where behavior 1 takes place absent any behavioral intervention. A behavioral spillover is empirically identified as the effect of the behavioral intervention in the treatment group on a subsequent, not targeted, behavior 2, compared to the corresponding change in behavior 2 in the control group. Unexpected spillovers and additional insights (e.g., drivers, barriers, mechanisms) are elicited through analysis of qualitative data. In the spirit of the pre-analysis plan, a systematic checklist is finally proposed to guide researchers and policy-makers through the main stages and features of the study design in order to rigorously test and identify behavioral spillovers, and to favor transparency, replicability, and meta-analysis of studies.

## Introduction

### What Does Spillover Offer?

Academic and policy interest in ‘behavioral spillover’ has grown considerably in recent years (e.g., Austin et al., 2011; Truelove et al., 2014; Nilsson et al., 2016). Spillover is where the adoption of one behavior causes the adoption of additional, related behaviors. As we discuss below, we assume that the initial behavior change is due to an intervention, although other definitions of behavioral spillovers do not assume this (Nash et al., 2017). From a policy or practitioner perspective, the notion of behavioral spillover is attractive because it appears to hold the promise of changing a suite of behaviors in a cost-effective manner with little regulation which might be politically unpopular. For many pressing social issues, such as climate change or obesity, spillover is thus a promising method of achieving the scale of lifestyle change required to address these, in contrast to the typically small-scale behavioral changes achieved from most individually focussed interventions (Capstick et al., 2014). From an academic perspective, spillover is intriguing because it sheds new light on the process of lifestyle change: rather than examining behavior change from the perspective of individual behaviors in isolation, spillover draws attention to the holistic relationships between behaviors within and between contexts, and hence refocus the researchers’ perspective on the complex behavioral ecologies that represent lifestyles (Geller, 2001; Schatzki, 2010).

A variety of empirical methods have been used to measure behavioral spillovers to date, from qualitative self-reports to statistical/econometric analyses, from online and lab experiments to field experiments. Detecting spillover has often proved challenging, and there is a need for both conceptual and methodological clarity in order to move the field forward. The aim of this paper is to critically review the main experimental and non-experimental methods to measure behavioral spillovers to date, and to discuss their methodological strengths and weaknesses. A consensus mixed-method approach is then discussed which uses between-subjects randomization and behavioral observations together with qualitative self-reports in a longitudinal design in order to follow up subjects over time. We conclude by proposing a systematic checklist to guide researchers and policy-makers through the main stages and features of the study design in order to rigorously test and identify behavioral spillovers, and to favor transparency, replicability, and meta-analysis of studies.

### Definition of Behavioral Spillover

The term ‘spillover’ has been applied to a wide variety of phenomena, including the spread of knowledge, attitudes, roles/identities, or behaviors from a given domain (e.g., health, environment, care-giving), group, or location, to a different domain, group or location (e.g., Geller, 2001; Poortinga et al., 2013; Littleford et al., 2014; Rodriguez-Muñoz et al., 2014; Poroli and Huang, 2018). The main appeal of such broad definition of behavioral spillover is that it encompasses a rich variety of spillover effects at both a micro and a macro level which are of key interest for policy and practice purposes, such as cross-domains, inter-personal, and cross-regional spillover effects of phenomena and interventions. However, the processes underpinning these diverse effects are highly heterogeneous, ranging from cognition (e.g., learning, problem-solving) and self-regulation, through interpersonal effects (e.g., modeling, contagion) to individual behavior change, and there is little these processes have in common besides the idea of (often unanticipated) diffusion of some effect.

In what follows, we assume a narrower and more specific definition of behavioral spillover that matches more closely the methodological approach that we have in mind. In particular, behavioral spillover can be defined as the observable and causal effect that a change in one behavior (behavior 1) has on a different, subsequent behavior (behavior 2). More specifically, to constitute behavioral spillover, the two behaviors must be different (i.e., not related components of a single behavior), sequential (i.e., behavior 2 follows behavior 1), and sharing, at a conscious or unconscious way, an underlying motive (i.e., an overarching goal or a ‘deep preference,’ such as, for example, pro-environmentalism or a healthy life) (Dolan and Galizzi, 2015; Nash et al., 2017). This concept of spillover has been examined in relation to different domains (safety, environment, health, finances, etc.) for some decades, although these effects have previously been labeled in diverse ways, including ‘response generalization’ (Ludwig and Geller, 1997; Geller, 2001), ‘the foot in the door effect’ (Freedman and Fraser, 1966; Beaman et al., 1983), and ‘moral licensing’ (Blanken et al., 2015; Mullen and Monin, 2016). We have conducted a systematic review of the literature (see Appendix for full details) and found that a total of 106 studies to date have used the above, more specific, definition of behavioral spillovers.^1^

Behavioral spillovers can be categorized as ‘promoting,’ ‘permitting,’ ‘purging,’ or ‘precipitating,’ as illustrated in Table 1.

**Table 1:** Types of behavioral spillovers (adapted from Dolan and Galizzi, 2015: no copyright permissions are required for the reproduction of this table): examples from health behavior.

	Behavior 2		
Behavior 1		*Eat healthily*	*Eat less healthily*
	*A run after work*	**Promoting**	**Permitting**
		I ran an hour, let’s keep up the good work	I ran an hour, I deserve a big slice of cake
	*Sofa-sitting after work*	**Purging**	**Precipitating**
		I’ve been lazy today, best not eat so much tonight	I’ve been lazy today, so, what the heck, let’s have a big slice of cake

Other real world examples from environmental behavior are whether a behavioral intervention to monetarily incentivize household waste separation has a significant effect not just on waste separation (behavior 1), but also on green shopping, traveling, and support to environmental policies (behavior 2), for instance (Xu et al., 2018a); or whether an intervention to restrict irrigation has a significant impact not just on water conservation (behavior 1), but also on recycling behavior (behavior 2), for example (Sintov et al., 2019).

The mechanisms thought to explain promoting or positive spillovers vary by discipline and theoretical framework. Psychological approaches have focussed particularly on two mechanisms: (a) self-perception, identity, or preference for consistency (behavior 1 changes how one sees oneself and the desire to act consistently with that self-image leads to behavior 2) and (b) self-efficacy, knowledge, or self-motivation/empowerment (satisfactorily undertaking behavior 1 increases confidence and perceived efficacy of action, motivating change in behavior 2; Nash et al., 2017). Permitting or negative spillovers have been typically explained in terms of moral licensing, whereby a virtuous initial behavior licenses or ‘permits’ a second indulgent or morally questionable behavior, or by a contribution ethic whereby an initial behavior justifies subsequent inaction (e.g., Thøgersen, 1999; Karmarkar and Bollinger, 2015). Rebound effects are a related phenomenon, studied more from an economic than psychological perspective, and describe increased energy consumption due to technical efficiency gains, thereby offsetting energy savings achieved (e.g., Sorrell et al., 2009).

Evidence for spillover remains somewhat mixed, with some studies finding effects under certain conditions that are not replicated in other studies (Nash et al., 2017). Conceptually, spillover remains defined and explained in a variety of ways, and there remain considerable gaps in understanding (e.g., the role of social processes, such as norms, in spillover; Nash et al., 2017). Methodologically, there is also no coherent approach to researching spillover, which may in part explain the mixed and inconsistent empirical results, and critically highlights a need to improve the rigor and transparency of spillover research.

### Overview of Spillover Research Methods and Measurement

A growing stream of the literature at the interface between economics and psychology is currently investigating ‘behavioral spillovers’ in (and across) different domains, including health, environmental, and pro-social behaviors. To date, there have been a variety of methods applied to studying spillover (see Table 2). These range from qualitative retrospective self-reports using biographical interviews (e.g., Nash et al., 2019) to controlled laboratory experiments with randomization to condition (e.g., Van der Werff et al., 2014a,b). Each approach offers different strengths and weaknesses. For example, qualitative approaches are able to elucidate unexpected spillovers and additional insights (e.g., drivers, barriers, mechanisms) not anticipated or measured in quantitative approaches. On the other hand, quantitative approaches allow for more measurement standardization and potentially for generalization, as well as affording insights into factors shaping behavior that individuals may be unable or unwilling to reflect on consciously through self-report.

**Table 2:** Overview of methods used to research behavioral spillover: examples from environmental behavior.

Methodological approach	Data collection and analysis methods	Examples from environmental behavior	Strengths	Weaknesses
Qualitative	• Interviews or open-ended survey questions	Austin et al., 2011; Boström et al., 2015; Nash et al., 2019; Uzzell and Räthzel, 2018	• Expose unexpected spillovers	• Risk of presentational bias
	• Thematic, content, discourse (or similar) analysis		• Shed light on spillover mechanisms, drivers and barriers	• Partial or selective recollection • No measurement standardization
	• Self-reports or other (e.g., practitioner) accounts			
	• Biographical (retrospective) or evaluative (during/immediately after intervention)			
Quantitative (cross-sectional)	• Survey, card sort or secondary data analysis (e.g., retail data)	Thøgersen, 1999; Barr et al., 2010; Whitmarsh and O’Neill, 2010; Austin et al., 2011; Gabe-Thomas et al., 2016	• Quantify strength of relationships between measured behaviors	• No causal relationships identified
	• Cluster or factor analysis		• Measurement standardization	• Limited to expected spillovers
	• Correlational analysis			
	• Regression analysis			
Quantitative (longitudinal)	• Surveys at 2+ timepoints	Thøgersen and Noblet, 2012; Kaida and Kaida, 2015; Poortinga et al., 2013; Thomas et al., 2016.	• Quantify strength of relationships between measured behaviors	• No causal relationships identified
	• Repeated measures analysis or multi-level modeling		• Measurement standardization	• Limited to expected spillovers
	• Correlational analysis			
	• Regression analysis (including time series, panel data, and difference-in-difference models)			
Quantitative (experimental)	• Online, laboratory, or field experiments	Van der Werff et al., 2014a,b; Juhl et al., 2017.	• Causal relationships and mechanisms identified	• Limited to expected spillovers
	• Self-reported or observed behavior		• Measurement standardization	
	• Randomization to behavioral intervention			
	• Analysis of variance			
	• Regression analysis			
Mixed-methods	• Combination of qualitative and quantitative methods (e.g., experiment and interviews)	Verfuerth, in preparation; Lede, in preparation.	As above	As above

Measurement of spillover has been undertaken in a variety of ways that reflect the range of methods used. Qualitative approaches tend to rely on self-reported accounts of behavior change; whereas quantitative approaches may use self-reports or observations of behavior. A key weakness in the literature to date, has been a reliance on self-reported behavior, which is known to be only weakly correlated with actual behavior (e.g., Kormos and Gifford, 2014). Furthermore, several studies claiming to find spillover have found change in behavioral intentions or attitudes following an initial behavior change, which is not strictly spillover (Van der Werff et al., 2014a). Few studies also conduct follow-up measurements, so the durability of any immediate spillover effects is unknown. There has also been a reliance on correlational or longitudinal designs which are unable to shed light on causal processes; and within longitudinal designs approaches differ in how to detect spillover (Capstick et al., submitted). Finally, there have also been few attempts to bring together quantitative and qualitative approaches, thus providing complementary insights and addressing respective weaknesses in approaches (Creswell, 2014). In the following section, we describe how spillover should be measured in experimental and non-experimental approaches that seeks to build on this literature and address limitations in the methods used to date.

## Measuring Spillover

We now turn from our observations of previous spillover research to a discussion of how we propose spillover research should ideally be conducted in order to reliably detect any spillover effects and expose mechanisms through which they may operate. Drawing on best practice in research design and reflecting principles of transparency and validity (e.g., Open Science Collaboration, 2015), we first discuss experimental studies, which elucidate causal mechanisms, and then non-experimental approaches, which afford other insights into spillover, as discussed above.

### How to Measure Behavioral Spillover: Experimental Studies

Rigorously designing and implementing randomized controlled experiments allows the researchers to obtain an unbiased estimate of the average treatment effect of a behavioral intervention (e.g., a ‘nudge,’ a monetary or non-monetary incentive, a ‘boost’ or ‘prime’). Because of sample selection bias, it is only by randomly assigning subjects to a treatment or to a control group that the researchers can identify the causal effect of a behavioral intervention on an observed outcome (Heckman, 1979; Burtless, 1995; Angrist and Pischke, 2009; List, 2011; Gerber and Green, 2012).

In practice, a variety of different randomized controlled experiments is available to researchers interested in testing behavioral spillovers. It is useful to refer here to the influential taxonomy of experiments in social sciences originally proposed by Harrison and List (2004): *conventional lab* experiments involve student subjects, abstract framing, a lab context, and a set of imposed rules; *artefactual field* experiments depart from conventional lab experiments in that they involve non-student samples; *framed field* experiments add to artefactual field experiments a field context in the commodity, stakes, task or information; and, finally, *natural field* experiments depart from framed field experiments in that subjects undertake the tasks in their natural environment, and subjects do not know that they take part into an experiment. The main idea behind *natural field* experiments is that the mere act of observation and measurement necessarily alters what is being observed and measured. In key areas of interest for behavioral spillovers, such as health, the environment or pro-social behavior, for instance, there are potential *experimenter demand effects* (i.e., participants change behavior due to cues about what represents ‘appropriate’ behavior for the experimenter: Bardsley, 2005; Levitt and List, 2007a,b; Zizzo, 2010); *Hawthorne effects* (i.e., simply knowing they are part of a study makes participants feel important and improves their effort and performance: Franke and Kaul, 1978; Adair, 1984; Jones, 1992; Levitt and List, 2011); and *John Henry effects* (i.e., participants who perceive that they are in the control group exert greater effort because they treat the experiment like a competitive contest and they want to overcome the disadvantage of being in the control group: Campbell and Stanley, 1963; Cook and Campbell, 1979).

Other, more recent, typologies of randomized controlled experiments are *online experiments* (Horton et al., 2011) conducted, for instance, using Amazon’s *Mechanical Turk* (*MTurk*) (Paolacci et al., 2010; Horton et al., 2011; Paolacci and Chandler, 2014); and *lab-field experiments* that consist of a first-stage intervention under controlled conditions (in the lab) linked to a naturalistic situation (in the field) where subjects are not aware that their behavior is actually observed. Lab-field experiments have been used to look at the unintended spillover effects of behavioral interventions in health (Dolan and Galizzi, 2014, 2015; Dolan et al., 2015), as well as at the spillover effects in terms of external validity of lab-based behavioral economics games of pro-social behavior (Galizzi and Navarro-Martinez, 2018).

Investigating experimentally the occurrence of behavioral spillover requires a mixed, longitudinal experimental design combining elements of between- and within-subjects design. Participants in an experiment are randomly allocated by the researcher either to a control group, or to (at least) one behavioral intervention group. In the control group (C), subjects are observed while they engage in a first behavior (behavior 1) and then in a different, subsequent, behavior (behavior 2). Each of the two subsequent behaviors is operationally captured and reflected into (at least) one corresponding outcome variable: B1 and B2. In practice, the choice of behavior 1 and behavior 2, as well as the choice of the corresponding outcome variables B1 and B2, is often based on theoretical expectations, previous literature, or qualitative evidence. It is also based on other, more pragmatic, considerations related, for example, to the ease of observing some specific positive or negative spillovers in the lab or the field, and to the ethical and logistical acceptability of changing some behaviors in an experimental setting. In what follows, we illustrate the measurement of behavioral spillovers in the simplest possible case of one single behavioral intervention group, and one single outcome variable for both B1 and B2. The extension to more complex cases is straightforward.

In the treatment group (T), a behavioral intervention (e.g., a ‘nudge,’ a monetary or non-monetary incentive, a ‘boost’ or ‘prime’) is introduced to directly target behavior 1, thus affecting the outcome variable B1. The between-subjects design naturally allows the researcher to test the effects of the behavioral intervention on the targeted behavior 1, by directly comparing B1 across the control and the treatment groups, that is, by comparing B1C versus B1T.

The between-subjects design, together with the longitudinal dimension of the experiment, also allows the researcher to check if the behavioral intervention has a ramification effect on the non-targeted behavior 2, thus affecting the outcome variable B2. In particular, the outcome of behavior 2 in the control group (B2C) serves as the baseline level for the extent to which behavior 2 is affected by behavior 1 in the absence of any behavioral intervention targeting behavior 1 (B1C) (see Table 3).

**Table 3:** Experimental design and variables to test behavioral spillovers.

	Behavior 1	Behavior 2
Control group (C)	B1C	B2C
Treatment group (T)	B1T	B2T
Difference	ΔB1	ΔB2

In contrast, the outcome of behavior 2 in the treatment group (B2T) captures the extent to which behavior 2 is affected by the ‘perturbed’ level of behavior 1 as a consequence of the introduction of the behavioral intervention (B1T).

Therefore, by directly comparing B2T and B2C, the difference ΔB2 = B2T – B2C captures the positive or negative change in the outcome variable for behavior 2 which is directly attributable to the change in the outcome variable for behavior 1, ΔB1 = B1T – B1C, which, in turn, is causally affected by the introduction of the behavioral intervention. That is, ΔB2 = B2T – B2C captures the ‘knock on’ behavioral spillover effect of the behavioral intervention targeting behavior 1 on the non-targeted, subsequent behavior 2.

In terms of sizes and statistical significance, such spillover effects may not be significantly different from zero (ΔB2 = 0), may be significantly and positively different from zero (i.e., ΔB2 > 0), or, finally, may be significantly and negatively different from zero (i.e., ΔB2 < 0). If the two behaviors share one common underlying ‘motive’ (in the sense of Dolan and Galizzi, 2015, of some overarching goal or deep preference such as ‘being healthy,’ ‘being pro-environmental,’ or ‘being pro-social’) then the experimental findings may thus be interpreted as evidence of no behavioral spillovers (ΔB2 = 0), evidence of originating ‘promoting’ or ‘precipitating’ behavioral spillover (ΔB2 > 0) or, finally, evidence of ‘permitting’ or ‘purging’ behavioral spillover (ΔB2 < 0).

Such an experimental design also allows the researchers to estimate not only the sign and the statistical significance of the behavioral spillover effects, but also their size. In particular, by comparing the relative changes in the outcome variables for behavior 1 and 2 as effects of the introduction of the behavioral intervention, the ratio between the proportional change (ΔB2/B2C) and the proportional change (ΔB1/B1C) allows the researcher to estimate the ‘elasticity’ of the behavioral spillovers: in analogy with standard price elasticity concepts, the elasticity is defined as the percentage change in behavior 2 per unitary percentage change in behavior 1, that is 𝜀_BS_ = (ΔB2/B2C)/(ΔB1/B1C).

This, in turn, allows the researcher to conclude whether a behavioral intervention causes behavioral ramifications which are small or large compared to the directly targeted change in behavior. In case of permitting or purging behavioral spillovers (i.e., ΔB1 and ΔB2 having opposite signs), and provided that B1 and B2 share the same metrics (or provided that they feed into the underlying motive in a way that the relative sizes of their changes ΔB1 and ΔB2 are conceptually comparable), this can provide further evidence on whether the permitting or purging spillovers are compensating each other completely or only partially (e.g., ‘backfire’ or ‘rebound’ effects).

Two further considerations are in order here. First, the above described definition and framework to measure behavioral spillovers in an experimental setting is sufficiently general and comprehensive to nest as a special case the situation where the behavioral intervention consists of behavior 1 itself. For example, in the ‘question-behavior’ and ‘survey’ promoting spillover effects discussed in Dolan and Galizzi (2015), the behavioral intervention consists of randomly assigning subjects to a brief survey or questionnaire eliciting past health, environmental, or purchasing behavior (e.g., Fitzsimons and Shiv, 2001; Zwane et al., 2011; Van der Werff et al., 2014a). In such a case, in fact, the behavioral intervention in the treatment group merely consists of exposing subjects to behavior 1 (e.g., a survey) before behavior 2 takes place. In the control group, on the other hand subjects go through behavior 2 without being previously exposed to behavior 1. Also in this, simpler, special case, behavioral spillover is measured as ΔB2 = B2T – B2C, but in this case the behavioral spillover captures the positive or negative change in the outcome variable for behavior 2 which is directly attributable to the mere exposure of subjects to behavior 1 in the treatment group (which, in this case, coincides with the behavioral intervention).

Second, the decision about the timeframe is crucial for the measurement of behavioral spillovers. Following subjects over longer timeframes implies, naturally, that it is more likely that spillover effects are effectively detected (Poortinga et al., 2013). Considering substantially long timeframe (ideally a few weeks or even months after the end of the intervention) is desirable in order to be able to assess the durability of spillover effects. Considering even longer timeframes (ideally over 3 or 6 months after the end of the intervention) is particularly important to be able to detect the formation of new habits sustained over time (Lally et al., 2010), rather than a behavioral change that is only transient. In any case, in order to favor transparency and replicability of experimental results, it is crucial that the researchers pre-specify in advance the timeframe over which subjects are followed up over time. The timeframe, in fact, is a key point of the checklist that we propose below.

### How to Measure Behavioral Spillover: Non-experimental Quantitative Studies

An analogous strategy can be used in non-experimental settings along the line of the difference-in-difference empirical approach (e.g., Card, 1992, 1996; Card and Krueger, 1994, 2000; see more below). In particular, the researcher can exploit the variation occurring naturally in the field outside their control and can use some ‘natural experiment’ as an exogenous ‘intervention’ in order to identify the likely effect of such an exogenous change on the variables of interest, despite the fact that participants are not randomly assigned to a proper experimental intervention.

The exogenous variation occurring naturally in the field can be a change in policy, a natural ‘shock’ (e.g., a health shock, a natural disaster, a political shock, an economic shock), a life event (e.g., birth of a child, death of a relative, divorce, unemployment), a technological advance, a discontinuity in the availability or in the access of a resource or an infrastructure. The source of the exogenous variation can also be ‘cognitive’ or ‘behavioral,’ such as an exogenous change in attention or awareness, provided that there are convincing reasons to argue that such a source of variation is exogenous (rather than endogenous) to the occurrence of behavioral spillovers.

In the standard difference-in-difference approach, two areas (e.g., two regions, two countries, two schools, two hospitals), are compared before and after the occurrence of a natural event (e.g., a policy, a shock) affecting one area (T) but not the other one (C). Typically, the change of the outcome of behavior 1 before (t = 0) and after (t = 1) the natural event in the ‘control’ area B1C_t = 1_ – B1C_t = 0_ is compared over time to the analogous change in the ‘treatment’ area B1T_t = 1_ – B1T_t = 0_, in order to see whether the trends show any significant difference in differences across the two areas (i.e., if B1T_t = 1_ – B1T_t = 0_, is statistically significantly different from B1C_t = 1_ – B1C_t = 0_).

In principle, an analogous comparison can be made considering the outcome variable of behavior 2 (B2, instead of B1), to see whether the natural event also has ramifications on a different, subsequent behavior, far and beyond the initial change on behavior 1. Therefore, the researcher can compare the change over time of the outcome variable for behavior 2 before (t = 0) and after (t = 1) the natural event in the ‘control’ area B2C_t = 1_ – B2C_t = 0_ to the analogous change in the outcome variable for behavior 2 in the ‘treatment’ area B2T_t = 1_ – B2T_t = 0_, in order to see whether the trends show any significant difference in differences across the two areas (i.e., whether B2T_t = 1_ – B2T_t = 0_, is statistically significantly different from B2C_t = 1_ – B2C_t = 0_). Analogous considerations to the ones described above can be made here concerning the sign, significance, and size of the behavioral spillovers in a non-experimental setting (e.g., Claes and Miliute-Plepiene, 2018).

As mentioned above, our framework is sufficiently general and comprehensive to nest, as a special case, the situation where the ‘intervention’ in an experimental setting, or the ‘shock’ or exogenous variation in a non-experimental setting, consists of behavior 1 itself. In such a case, the difference-in-difference approach described above reduces to the comparison of the change in the outcome variable for behavior 2 in the ‘treatment’ area that has been exposed to behavior 1 (B2T_t = 1_ – B2T_t = 0_) with the analogous change in the ‘control’ area which has not been exposed to behavior 1 (B2C_t = 1_ – B2C_t = 0_).

The empirical strategy described above has been illustrated having in mind our specific definition of behavioral spillover proposed in section “Definition of Behavioral Spillover,” that is, the observable and causal effect that a change in one behavior (behavior 1) has on a different, subsequent behavior (behavior 2). Nonetheless, a corresponding strategy can be adapted to some of the instances encompassed by the broader definition of spillover reported at the beginning of section “Definition of Behavioral Spillover,” that is the impact that an intervention in a given domain (e.g., health, the environment), group, or location, has on a different domain, group or location. In principle, two locations (e.g., two countries), can be compared before and after the occurrence of a natural event (e.g., a natural phenomenon, an intervention) affecting one domain (e.g., the environment) in one area (T) but not in the other one (C). The researcher can compare not only the change over time of the outcome variable for the domain directly involved in the phenomenon or originally targeted by the intervention (e.g., the environment), but also the change over time of the outcome variable for a different domain (e.g., health). Considering the knock-on effects of the phenomenon or intervention on different groups or regions is also possible in principle, although in practice the empirical analysis would need to account for other underlying intra-groups or intra-regional differences between the ‘control’ and the ‘treatment’ areas.

### How to Study Behavioral Spillover: Qualitative and Mixed-Methods Studies

A different, but potentially complementary, approach to studying spillover involves using qualitative methods, such as interviews analyzed thematically (e.g., Boström et al., 2015; Dittmer and Blazejewski, 2016; Nash et al., 2017; Uzzell and Räthzel, 2018; Thomas et al., 2019). As noted, such approaches have the advantage over quantitative approaches of exposing unexpected spillovers, as well as the shedding light on the drivers, barriers and mechanisms of spillover, and on participants’ experience and meanings associated with spillover. For example, Uzzell and Räthzel (2018) used life history interviews to examine how equivalent practices (as well as identities and meanings) develop over time and may be transferred between work and home; using diachronic and synchronic analyses allowed them to identify drivers and barriers to consistency of actions across time, as well as across contexts. Verfuerth et al. (2018) used depth interviews to explore the impacts of a workplace meat reduction intervention, and found unanticipated spillover across behaviors (e.g., to avoiding food waste) and contexts (to home); while Schütte and Gregory-Smith’s (2015) semi-structured interviews exposed cognitive and emotional barriers to pro-environmental spillover between home and holiday.

As such, qualitative methods provide valuable insight in their own right into spillover phenomena, but can also be combined with quantitative approaches in mixed-methods designs to address quantitative limitations (Verfuerth and Gregory-Smith, 2018). Various approaches can be used to ensure the quality of qualitative data, such as member validation (i.e., asking participants to check researcher interpretations), inter-rater reliability of coded data (i.e., using multiple coders and resolving any disagreement in interpretation), and reflexivity (i.e., fully documenting the processes used to collect data and the role and background of the researcher; Breakwell et al., 2012). Others have noted that the diversity of qualitative methods requires a range of criteria for assessing quality and validity (Reicher, 2000); but most agree at least that transparency and consistency are key (Braun and Clarke, 2006). The importance of being systematic is therefore a criterion of quality shared by both quantitative and qualitative methods.

A growing literature advocates the use of mixed-methods approaches in order to triangulate and provide complementary insights. Despite associations of qualitative and quantitative methods with divergent epistemological and ontological paradigms (Blaikie, 1991), this should not imply that qualitative and quantitative methods are essentially incommensurate (Bryman, 1988). Rather, the distinction between particular qualitative and quantitative methods can be understood as primarily technical, and not necessarily philosophical. Qualitative and quantitative methods offer different insights into spillover and each is better suited to answering different types of research question (e.g., What are the range of effects of an intervention? How is the development of identity and practices experienced over time and contexts? What causes and mediates spillover?). Thus, the rationale for combining methods stems from “the basic and plausible assertion that life is multifaceted and is best approached by the use of techniques that have a specialized relevance” (Fielding and Fielding, 1986, p. 34). Furthermore, using multiple methods allows interesting lines of inquiry exposed through one method to be explored further through another (Whitmarsh, 2009). At the same time, however, it is not assumed that aggregating data sources can provide a complete or ‘true’ picture of the social world (Silverman, 2001). Indeed, “the differences between types of data can be as illuminating as their points of coherence” (Fielding and Fielding, 1986, p. 31), for example leading to a re-examination of conceptual frameworks or assumptions (Tashakkori and Teddlie, 2003). The distinct challenges of researching spillover imply both qualitative and quantitative approaches are warranted to address different facets of the problem.

Mixed-methods designs may be sequential or concurrent, or both (Creswell, 2014). In the case of spillover studies, a mixed methods design might start with an initial qualitative and/or correlational phase to identify clusters of co-occurring behaviors which may indicate spillover, for which candidate behaviors (B1, B2, etc.) and the causal pathways connecting them can be examined in a subsequent experimental design, as outlined above. In addition, qualitative methods can be used alongside quantitative behavioral measures within the intervention phase to explore the experience, perceptions, and subjective wellbeing implications of the intervention, and to expose potentially unexpected spillover effects, as well as possible drivers, barriers, mechanisms, and mediating/moderating factors for any spillover. This might take the form of interviews with a sub-sample of experimental participants, or one or more open-ended questions in a post-intervention survey. Where spillover is detected through quantitative experimental methods, qualitative data may help explain why this effect has occurred, and how this has been subjectively perceived and experienced. In the event that spillover is not detected via the experimental methods outlined above, qualitative methods may explain why not, or they may expose other, unquantified spillover effects. Qualitative, quantitative, and experimental methods should thus be seen as complementary, rather than substitute, empirical methods to explore and assess behavioral spillovers. So far, there exist few mixed-methods studies of spillover, but those that have been undertaken appear to demonstrate that a mixed methodology can elucidate multiple aspects of spillover processes and experiences (Barr et al., 2010; Verfuerth et al., 2018; Thomas et al., 2019).

## A Practical Checklist

Exploring and detecting behavioral spillovers is a research and policy task which should be undertaken using a systematic and transparent approach, in the same spirit of, and closely in line with, the recent best practices favoring and advocating systematization and transparency in psychological and behavioral sciences (Ioannidis, 2005; Higgins and Green, 2011; Simmons et al., 2011; Miguel et al., 2014; Simonsohn et al., 2014; Open Science Collaboration, 2015; Munafò et al., 2017). In the previous section, we outlined how this might be achieved using different research designs.

Abstracting from these exemplar designs, here we propose a checklist of points which should be explicitly stated and addressed by the researcher prior to undertaking of experimental and empirical analysis. The 20-item checklist is in line with, and in the same spirit of, other checklists designed to systematically assess the methodological quality of prospective studies, for example by the Cochrane Collaboration (Higgins and Green, 2011). The checklist is also in line with, and in the same spirit of, other more general checklists guiding researchers through pre-registration of studies and pre-analysis plans (e.g., the Open Science Framework^2^). Once filled in, the checklist for a prospective study should be deposited in a dedicated website which is going to be launched with the publication of this special issue, and which will be available at: https://osf.io/9cqjf/. The website will also include a data template where data from deposited studies could be shared, collated, and combined in order to conduct collaborative systematic reviews and meta-analyses of the literature.

The 20 questions of the checklist are below. In what follows we briefly illustrate each question with a real case study, the recent study by Xu et al. (2018a) on household waste separation:

1. What are the setting and population of interest?
      •Four geographically adjacent communities in the Yuhang District of Hangzhou, Zhejiang Province, China.
2. Is this an experimental or a non-experimental study?
      •An experimental study (a framed field experiment).
3. If this is a non-experimental quantitative study, what is the empirical identification strategy (e.g., difference-in-difference)?
      •N/A.
4. If this is a quantitative study, what is the control group?
      •The control group were participants in each community who were not exposed to any formal promotion of waste separation.
5. How have the behaviors been selected (e.g., existing literature, qualitative evidence)?
      •Based on previous findings and on the literature.
6. What is the targeted behavior 1?
      •Sorting daily garbage and bringing it to waste collection sites.
7. What are the outcome variables for behavior 1 (i.e., how will you measure behavior 1)? (Please list them and briefly describe each outcome variable, indicating whether this is directly observed or self-reported behavior.)
      •Difference in self-reported household waste collection before and after the interventions.
8. How many intervention groups there are?
      •Originally there were three intervention groups, but one condition (‘mixed condition’) was then excluded (see footnote 1 in page 28).
9. What are the behavioral interventions targeting behavior 1? (Please list them and briefly describe each of them.)
      •In the Environmental Appeal (EA) condition participants were given 3 monthly 30-min presentations where they were informed about the environmental benefits of waste separation. In the Monetary Incentive (MI) condition participants were given 3 monthly 30-min presentations where they were informed that they could earn ‘green scores’ from a recycling firm if they sorted their daily garbage and brought it to waste collection sites. In the ‘mixed condition’ participants were given 3 monthly 30-min presentations where they were informed of both EA and MI (this condition was later excluded from the analysis).
10. What is the non-targeted behavior 2?
      •A set of 25 self-reported environmental behaviors or self-reported willingness to engage in environmental behaviors, including both ‘private-sphere’ behaviors (e.g., green shopping, traveling) and ‘public-sphere’ behaviors (e.g., support to environmental policies, environmental citizenship actions).
11. What are the outcome variables for behavior 2 (i.e., how will you measure behavior 2)? (Please list them and briefly describe each outcome variable, indicating whether this is directly observed or self-reported behavior.). If there are multiple outcome variables for behavior 2, does the study correct for multiple hypotheses testing? (Please describe which correction is used.)
      •All the outcome variables for the 25 environmental behaviors or willingness to engage in environmental behaviors are self-reported, and are collected by a monthly survey. There is no explicit correction for multiple hypotheses testing.
12. What is the expected underlying motive linking behavior 1 and behavior 2?
      •Pro-environmental identity (page 28).
13. What are the expected mechanisms moderating and/or mediating the changes in the outcome variables for behavior 2?
      •The expected mechanisms are both promoting/positive behavioral spillovers such as the activation of a stronger pro-environmental identity, and permitting/negative behavioral spillovers such as moral licensing (page 28). Pro-environmental identity and environmental concern are expected to mediate promoting/positive spillovers. Relief of guilt is expected to mediate permitting/negative spillovers.
14. What is the expected time frame during which behavioral spillovers will be tested, and during which the durability of spillover and habit formation will be assessed?
      •The expected time frame is not explicitly mentioned, but participants are followed up for 3 months.
15. What is the expected participant attrition between behavior 1 and behavior 2?
      •There is no explicit discussion of expected attrition. However, attrition was not only high, but it was asymmetric across different conditions. At the end of the experiment (3 months after), only 195 out of the 400 participants originally recruited remained in the study: 80 (out of 100) in the EA group, 36 (out of 100) in the MI group, and 79 (out of 100) in the control group (all the 100 participants in the mixed condition group were excluded).
16. What is the expected direction of the changes in the outcome variables for behaviors 1 and 2 between the intervention groups and the control group (i.e., are positive or negative spillovers expected)?
      •Both promoting/positive and permitting/negative spillovers were expected (page 28).
17. What are the expected sizes and standard errors of the changes in the outcome variables for behaviors 1 and 2 between the intervention groups and the control group?
      •There is no explicit discussion of the expected effect size or standard errors of the changes in the outcome variables for behaviors 1 and 2.
18. What is the minimum expected sample size to test and detect the occurrence of behavioral spillover?
      •The study recruits n = 100 participants in each of the four groups, but there is no explicit justification of the minimum expected sample size to test and detect the occurrence of behavioral spillovers.
19. If collecting qualitative data, how will the quality of this data be ensured and assessed (e.g., reflexivity, consistency)?
      •A number of psychological constructs were collected (including four items to measure personal identification with environmental protection; three items to measure personal concern for the environment, ecology, and the earth; three items to measure feelings of disappointment, guilt, and regret for past environmentally unfriendly behaviors) and used in exploratory factor analysis, but no further qualitative data was collected.
20. If using mixed-methods approaches, how will insights from different methods be combined?
      •N/A.

## Conclusion

We have critically reviewed the main methods to measure behavioral spillovers to date, and discussed their methodological strengths and weaknesses. We have proposed a consensus mixed-method approach which uses a longitudinal between-subject design together with qualitative self-reports: participants are randomly assigned to a treatment group where a behavioral intervention takes place to target behavior 1, or to a control group where behavior 1 takes place absent any behavioral intervention. A behavioral spillover is empirically identified as the effect of the behavioral intervention in the treatment group on a subsequent, not targeted, behavior 2, compared to the corresponding change in behavior 2 in the control group.

In the spirit of the pre-analysis plan, we have also proposed a systematic checklist to guide researchers and policy-makers through the main stages and features of the study design in order to rigorously test and identify behavioral spillovers, and to ensure transparency, reproducibility, and meta-analysis of studies.

While ours is arguably the first methodological note on how to measure behavioral spillovers, it has of course limitations. The main limitation is that our experimental and empirical identification strategy relies on our specific definition of behavioral spillover – i.e., the observable and causal effect that a change in one behavior (behavior 1) has on a different, subsequent behavior (behavior 2). As mentioned in section “Definition of Behavioral Spillover,” broader definitions of spillover exist that can encompass attitudinal change, learning, interpersonal influences, and other disparate processes. While we have suggested here that a similar approach to ours (i.e., longitudinal mixed-methodology) might apply in these cases, there may be also be methodological considerations specific to each type of spillover that warrants its own methodological checklist. Even applying our more specific definition of behavioral spillover, it would be possible to define alternative methodological checklists that, for example, apply solely quantitative or qualitative methods (cf. Uzzell and Räthzel, 2018). However, as we have argued, we believe there is benefit in combining methods as they can offer different insights or address different research questions relating to spillover.

We would like to conclude by briefly mentioning a few other directions where we envisage promising methodological developments in the years to come. First, the current technological landscape naturally lends itself to a systematic measurement of behavioral spillovers in a variety of research and policy domains. Today an unprecedented richness of longitudinal data are routinely collected at an individual level in terms of online surveys, apps, smart phones, internet of things (IoT) and mobile devices, smart cards and scan data, electronic administrative records, biomarkers, and other longitudinal panels. This is creating, for the first time in history, an immense potential for following up individuals across different contexts and domains, and over time, for months, years, and even decades. This new technological landscape is also creating previously unexplored opportunities for ‘behavioral data linking,’ that is, for the linkage of behavioral experiments with other sources of longitudinal data (Galizzi, 2017; Galizzi et al., 2017; Galizzi and Wiesen, 2018; Krpan et al., 2019). On the one hand, the scope for systematically testing the occurrence of behavioral spillovers using rigorous empirical and experimental methods is therefore enormous. On the other hand, the endless wealth of research hypotheses, outcome variables, and data points makes even more important for researchers to embrace the best practices discussed above in order to ensure transparency, openness, and reproducibility of science.

Second, a promising methodological line of research about behavioral spillover concerns the rigorous investigation of the factors mediating and moderating the occurrence of behavioral spillover, for example in terms of accessibility (Sintov et al., 2019). Further work in this direction is likely to develop also thanks to the triangulation of different sources of data enabled by the above described shift in the technological landscape.

All these future developments reinstate the importance of developing a collective discussion about clear and transparent methodological guidelines to measure behavioral spillovers. We hope that with the present article we have contributed to at least start such a discussion. The time is ripe to foster a collaborative endeavor to systematically test behavioral spillovers across all research and policy domains, contexts, and settings.

## Author Contributions

MG initiated and led the paper writing. LW contributed to paper writing.

## Conflict of Interest Statement

The authors declare that the research was conducted in the absence of any commercial or financial relationships that could be construed as a potential conflict of interest.

## Figures and Tables

**Figure A1:**
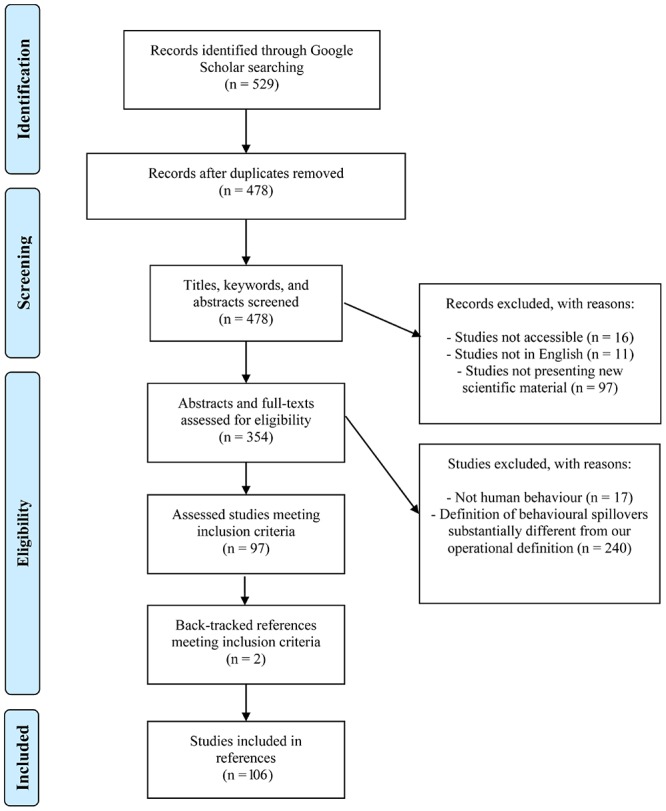
PRISMA flow diagram of systematic review.

## Footnotes

1 These 106 studies are: Bratt, 1999, Thøgersen, 1999, Hertwich, 2005, Karremans et al., 2005, Cornelissen et al., 2008, Hecht and Boies, 2009, Sorrell et al., 2009, Zimmerman, 2009, Savikhin, 2010, Sheremeta et al., 2010, Dickinson and Oxoby, 2011, Nolan, 2011, Bednar et al., 2012, 2015, Cason et al., 2012, Thøgersen and Noblet, 2012, Xanthopoulou and Papagiannidis, 2012, Alpizar et al., 2013a,b, Baca-Motes et al., 2013, Cason and Gangadharan, 2013, Falk et al., 2013, Godoy et al., 2013, Juvina et al., 2013, Norden, 2013, Poortinga et al., 2013, Savikhin and Sheremeta, 2013, Swim and Bloodhart, 2013, Tiefenbeck et al., 2013, Bech-Larsen and Kazbare, 2014, Dolan and Galizzi, 2014, 2015, Lanzini and Thøgersen, 2014, Littleford et al., 2014, Spence et al., 2014, Tiefenbeck, 2014, Truelove et al., 2014, 2016, Van der Werff et al., 2014a,b, Goswami and Urminsky, 2015, Kaida and Kaida, 2015, Karmarkar and Bollinger, 2015, Lacasse, 2015, 2016, 2017, Schütte and Gregory-Smith, 2015, Steinhorst et al., 2015, Zawadzki, 2015, Banerjee, 2016, Dittmer and Blazejewski, 2016, Eby, 2016, Gholamzadehmir, 2016, Ha and Kwon, 2016, Lauren et al., 2016, 2017, Margetts and Kashima, 2016, Nilsson et al., 2016, Polizzi di Sorrentino et al., 2016, Steinhorst and Matthies, 2016, Suffolk, 2016, Suffolk and Poortinga, 2016, Thomas et al., 2016, 2019, Carpenter and Lawler, 2017, Carrico et al., 2017, Crookes, 2017, Fenger, 2017, Galbiati et al., 2017, Hedrick et al., 2017, Jessoe et al., 2017, Juhl et al., 2017, Kesternich et al., 2017, Klein, 2017, Krieg and Samek, 2017, McCoy and Lyons, 2017, Nash et al., 2017, 2019, Werfel, 2017, Xie et al., 2017, Angelovski et al., 2018, Bednar and Page, 2018, Chatelain et al., 2018, Claes and Miliute-Plepiene, 2018, Dutschke et al., 2018, Ghesla et al., 2018, Lawler, 2018, Liu et al., 2018, Panos, 2018, Peters et al., 2018, Santarius and Soland, 2018, Schmitz, 2018, Seebauer, 2018, Shreedhar, 2018, Shreedhar and Mourato, 2018, Tippet, 2018, Vasan, 2018, Verfuerth and Gregory-Smith, 2018, Vincent and Koessler, 2018, Whitmarsh et al., 2018, Xu et al., 2018a,b, Capstick et al., 2019, Fanghella et al., 2019, Krpan et al., 2019, Sintov et al., 2019.

2 https://osf.io/

## Appendix: Methodology of Systematic Review of the Literature

In conducting and reporting our systematic review of the literature, we followed as closely as possible the Preferred Reporting Items for Systematic Reviews and Meta-Analyses (PRISMA) checklist and guidelines (Moher et al., 2009), as explained below.

### Search Strategy and Key Terms

Google Scholar was searched in December 2018 using the following combinations of exact phrases in the advanced search settings:

(1) “behavioral spillover” (field TX all text) OR
(2) “behavioral spillover” (field TX all text).

### Selection and Exclusion Criteria

The authors reviewed and assessed all the references systematically, following a two-stage strategy. In the first stage, the inclusion criteria were applied to the title, the keywords, and the abstract; in the second stage, the criteria were applied to the abstract and the full text. All the papers were independently assessed for inclusion by each of the authors. Differences in opinions between the authors were solved through discussion.

The two stages worked as follows. In the first stage, a study was included only if it satisfied the following three criteria:

(1) The study was available (no broken link).
(2) The study was written in English.
(3) The study presented new scientific material, in terms of: new empirical evidence or original experimental analysis of behavioral spillover; new theoretical definitions or conceptual frameworks for behavioral spillovers; systematic reviews or meta-analyses of existing studies on behavioral spillovers. This criterion excluded non-systematic reviews, commentaries, editorials, letters, or similar items.
      Each article was sequentially evaluated against the three criteria, starting with criterion one and ending on criterion three. Whenever a criterion was not met, the article was excluded.
      In the second stage, the abstract and the full text of the studies shortlisted in the first stage were screened, evaluated, and finally included according to two further criteria:
(4) The study considered human behavior.
(5) The study used a definition of behavioral spillover substantially in line with our operational definition in section “Definition of Behavioral Spillover,” that is, the observable and causal effect that a change in one behavior (behavior 1) has on a different, subsequent behavior (behavior 2).

We included both published and unpublished studies, for example studies in working paper or in dissertation form. If both published and unpublished versions of the study were available, we considered the published version. If different dates of the unpublished versions were available, we considered the most recent one.

To ensure that the set of studies retrieved was exhaustive and comprehensive, for each included study, we also back-tracked and screened all the references cited in the article, applying the same inclusion criteria explained above.

### Search Results

The initial Google Scholar search resulted in a total number of *n* = 529 entries on December 17th, 2018 (*n* = 305 for “behavioral spillovers” and *n* = 224 for “behavioral spillovers.” After *n* = 51 duplicates were removed, the resulting number of studies was *n* = 478. We then excluded the papers that were not accessible (*n* = 16), were not written in English (*n* = 11), or did not present new scientific material (*n* = 97). A total of *n* = 354 studies met all three criteria in this first stage of our selection strategy.

The abstract and the full text of the *n* = 354 studies shortlisted were then screened and evaluated. We then excluded the studies that did not focus on human behavior (*n* = 17), and the studies whose definitions of behavioral spillovers was substantially different from our operational definition – or which did not define behavioral spillovers at all (*n* = 240). A total of *n* = 97 studies matched all the inclusion criteria in this second stage.

Back-tracking, screening, and evaluating the references cited in these *n* = 97 articles against the same inclusion criteria retrieved further *n* = 9 studies. So, at the end of the whole process, the systematic review resulted in a total of *n* = 106 selected studies.

Of the *n* = 106 selected studies, *n* = 12 are Doctoral theses, *n* = 5 are Master theses, and *n* = 12 are still unpublished works, all which shows the growing interest on behavioral spillovers.

The selection process and the number of papers excluded and included in each stage are summarized in the PRISMA flow chart in Figure A1.
